# The Institutional Library

**Published:** 1921-03-05

**Authors:** 


### THE INSTITUTIONAL LIBRARY.

**Memories and Musings of a Hospita Surgeon** (Maclehose, Jackson & Co. 1920. Price 7s. 6d. net.)

In an ideal book fulfilling the promise of a title such as this, anonymity might well be a source of additional interest by stimulating the reader to make a guess, from internal evidence, as to the author—or, at least, to draw a mental picture of his character and personality. Unfortunately this book displays the author's weaknesses rather than his strength. The preface strikes a note of pompous egotism which one hoped was merely accidental, the result of his very obvious inexperience of authorship: but he never gets away from it, and a total lack of humour further handicaps him all through. That a man can spend forty-seven and a-half years of professional life at a hospital and produce homilies as vapid and uninteresting as these chapters is disappointing, indeed humiliating; but so it is, and the least said about it, the soonest mended. Will not some hospital surgeon or physician with as long an experience, plus some of the attributes of "Peter Harding," undertake something on similar lines to show how this sort of thing ought to be done?

**General Practice and X-Rays.** By Alice V. Knox, M.B. (Edinburgh Medical Series. A. & C. Black. Pp. 214. Price 15s. net.)

THIS monograph, without going into any general discussion of technique and methods, aims at defining for the general practitioner the various uses of Röntgen rays and the ways in which they can be used, both for diagnosis and in therapeutics. This aim is very fully and satisfactorily fulfilled; the author is evidently enthusiastic as well as expert in this branch of medical science, and the only note of dissent which she is likely to hear will be, we fancy, that the limitations of *x*-rays are in certain directions insufficiently recognised. On the other hand, she is rightly emphatic on the paramount importance of expert radiography in many diseases at an early stage. She insists on the use of "radiogram" to describe the photographs produced by radiography, in preference to the incorrect "radiograph"; but Dr. Robert Knox, who contributes a section on apparatus, etc., will not conform to this usage, and sticks to "radiograph." The "radiograms," call them what one will, are numerous and well reproduced: which accounts, doubtless, for the high cost of the book. Fig. 3, by the way, is described as a negative, but appears to be a positive. The whole work carries out very fully and satisfactorily the objects with which it has been written.

**Atlas of the Sensory Cutaneous Nerves.** By WILLIAM IBBOTSON, M.R.C.S., L.R.C.P. (London: The Scientific Press, Ltd., 28-29 Southampton Street, Strand, London, W.C. 2. Price 7s. 9d. net.)

THE author of this little handbook is to be congratulated upon the eminently practical manner in which he has presented a branch of anatomy which is a source of considerable difficulty to the average student. In a series of excellent coloured diagrams, each with explanatory notes tabulated in a simple and concise manner, he has demonstrated the cutaneous nerve supply of the whole of the body. So good, indeed, are the diagrams that any one with a slight knowledge of the subject could readily refresh his memory by a glance at them without the aid of the notes. By the student who realises the difficulty of memorising a mass of prosaic though nevertheless important facts by time-worn methods which destroy the pleasure of study, the atlas will certainly be welcomed. Its usefulness need not, however, be limited to the student. Few practitioners of medicine find it possible to remember all the details of the nervous supply of the body, a knowledge of which is essential to accurate diagnosis in cases of nerve lesion; to these the atlas should prove invaluable as an aid to memory, and avoid much waste of time. The atlas is neatly bound in a red cloth cover. The letterpress is good, and the paper of a quality which adds to the brilliance of the plates.

**Elements of Medicine.** By the late ALFRED H. CARTER, M.D., M.Sc. Edited by Dr. A. G. GIBSON, of Oxford (H. K. Lewis & Co., Ltd.)

WE have read with pleasure the latest edition of this well-known book. The book follows its original form, and parts have been rewritten to enable it to be an accurate index of modern ideas in medicine. In the sections dealing with diseases of the nervous system we would have welcomed a few diagrams—not microphotographs—to indicate to the student the exact position of the pathological lesion. The therapeutic index, if taken only as a guide, we think is very useful for students who are trying to put into practice the knowledge gained from their pharmacology text-books. The form of the book is one that we have always recommended to students entering the wards, as it paves the way for, and is never meant to supplant, the larger text-books of medicine.

**The Medical Examination of Airmen.** By Dr. MAUBLANC and Dr. RATIÉ. Translated from the French. (Bale, Sons, & Danielsson. Pp. 8. Price 5s. net.)

This elementary book on the selection of the right and the rejection of the wrong types from amongst the candidates for training as air-pilots concerns itself with military, as opposed to civilian, conditions. It is a careful and exact piece of work, essentially practical in character. The introduction to English readers by a Wing Commander of our own R.A.F. medical service, promised on the title-page, is not to be found in the text, presumably by an oversight in the printing or binding.

**Normal Histology:** WITH SPECIAL REFERENCE TO THE STRUCTURE OF THE HUMAN BODY. By GEORGE A. PIERSOL, M.D., Sc.D. Eighth Edition. Philadelphia and London: J. B. Lippincott Company. Price 21s.)

THE eighth edition of this text-book, in general production, printing, and plate illustrations, is typical of the good work done by American publishers. For this reason alone we might well recommend the student to use this book as his histological companion, but there is another useful feature to be noted. The histological descriptions have been prefaced throughout with references to macroscopic features, a particularly valuable measure in view of the early position of histology in the medical curriculum and the inevitably limited knowledge of the student at this stage.

**Infant Weight Chart** (H. K. Lewis & Co. Price 2s. 6d. net.)

A FOLDING weight chart, 2 ft. by 20 in. when opened out, and providing space for graphic record of an infant's progress for the first six months of life, is a superfluity for most babies, though there are some for whom it would be helpful. The price at which this one is published is in any case prohibitive, and there can be few who will consent to pay so much.

